# Association Between Depressive Symptoms and Serum Brain-Derived Neurotrophic Factor Levels in Patients With First-Episode and Drug-Naïve Schizophrenia

**DOI:** 10.3389/fpsyt.2022.911384

**Published:** 2022-06-09

**Authors:** Yuxuan Wu, Xiangdong Du, Ruchang Yang, Yan Yue, Ruijie Peng, Siqi Wu, Haitao Wang, Yue Zhou, Xiaojia Fang, Nian Yuan, Ronghua Li, Jun Zhang, Siyun Zou, Xueli Zhao, Xiaoli Lyu, Zhe Li, Xiaobin Zhang, Xiangyang Zhang

**Affiliations:** ^1^Suzhou Medical College of Soochow University, Suzhou, China; ^2^Suzhou Guangji Hospital, The Affiliated Guangji Hospital of Soochow University, Suzhou, China; ^3^School of Psychology and Mental Health, North China University of Science and Technology, Tangshan, China; ^4^Xuzhou Medical University, Xuzhou, China; ^5^CAS Key Laboratory of Mental Health, Institute of Psychology, Chinese Academy of Sciences, Beijing, China

**Keywords:** schizophrenia, depressive symptoms, brain-derived neurotrophic factor, first episode psychosis, serum

## Abstract

Previous studies have revealed that brain-derived neurotrophic factor (BDNF) levels are inversely associated with the severity of depressive symptoms. In addition, serum BDNF levels tend to increase with improvement in depressive symptoms. There is also evidence that BDNF has a possible role in the pathophysiology of schizophrenia. Therefore, the purpose of this study was to determine whether BDNF levels correlated with depressive symptoms in patients with first-episode and drug-naïve (FEDN) schizophrenia. In this study, 90 patients with FEDN schizophrenia and 60 healthy controls were recruited. The Positive and Negative Syndrome Scale (PANSS) and the 17-item Hamilton Depression Scale (HAMD-17) were used to gage psychopathological and depressive symptoms, respectively. All participants had their BDNF levels measured using a sandwich enzyme-linked immunosorbent test. Serum BDNF levels were lower in patients with FEDN schizophrenia compared with healthy controls. Moreover, patients with depressive symptoms exhibited a higher PANSS total score and a higher general psychopathology score than those without depressive symptoms (*p* < 0.05). For patients with depressive symptoms, serum BDNF levels were higher than in those without depressive symptoms (*p* < 0.05). An association between BDNF levels and the positive subscore was also observed (*p* < 0.01). However, there was no significant association between BDNF levels and HAMD scores (*p* > 0.05). In conclusion, BDNF levels were shown to be higher in the serum of patients with FEDN schizophrenia with depressive symptoms than in those without. Additionally, low levels of serum BDNF may contribute to the positive symptoms of FEDN schizophrenia but not to depressive symptoms.

## Introduction

Depressive symptoms are common in patients with first-episode schizophrenia, not only concurrent with, but also preceding, the first episode of schizophrenia (1). The prevalence of depressive symptoms among patients with schizophrenia has been estimated to range between 30 and 70% (2). Patients with schizophrenia are more likely to experience depressive symptoms than the general population (3). In addition, depressive symptoms typically manifest as hallmarks of schizophrenia and are associated with worse prognoses for schizophrenia (4, 5). Therefore, the quest for molecular biomarkers of depressive symptoms in schizophrenia is vital for the development of effective therapies to limit the detrimental consequences of depressive symptoms.

Brain-derived neurotrophic factor (BDNF), one of the most extensively distributed neurotrophins in the adult mammalian brain, plays a key role in supporting the dendrites of various central nervous system (CNS) neurons, such as mood-regulating neurons (6–9). According to previous studies, BDNF is implicated in the pathophysiology of schizophrenia and is associated with schizophrenia-related phenotypes (10, 11). A number of studies have shown that the serum BDNF levels of patients with schizophrenia were lower than those of healthy controls (12–15). In addition, serum BDNF levels have a substantial correlation with the Positive and Negative Syndrome Scale (PANSS) score (16). Furthermore, the mRNA expression of BDNF and tropomyosin receptor kinase B (TrkB) was found to be reduced in the prefrontal cortex and hippocampus of patients with schizophrenia, indicating that BDNF should be studied as a potential target for antipsychotic medications (17, 18).

In addition to schizophrenia, altered levels of BDNF in the hippocampus and blood have been associated with other psychiatric disorders, including depression (19, 20). For example, several animal studies have shown that BDNF appears to have a crucial role in depressive-like behavior in rats (21, 22). In human research, the description of BDNF levels in peripheral blood may be traced back to the early work of Karege, who suggested that major depression was characterized by low serum BDNF levels (23). According to increasing evidence, the interaction of BDNF-TrkB and dopamine (DA) signaling in midbrain circuits plays a major role in the pathophysiology of depression (24–26). There is also evidence that serum BDNF levels are decreased in depressed patients and recover to normal following antidepressant therapy (27, 28).

The association between depressive symptoms and BDNF in patients with schizophrenia has received limited research attention, despite the fact that many studies have investigated the associations between BDNF and memory and cognitive performance in schizophrenia (20, 29–31). According to an earlier study, the BDNF Val66Met polymorphism has been reported to affect the intensity of depressive symptoms in patients with schizophrenia (32). In addition, after 12 weeks of olanzapine medication, researchers found an increase in serum BDNF levels and a decline in depressive symptoms (33). Although Noto et al. reported that individuals with more severe depressive symptoms exhibited higher levels of BDNF (34), no research has studied the relationship between comorbid depression and BDNF in patients with first-episode schizophrenia. We therefore set out to explore whether BDNF is associated with depressive symptoms in patients with first-episode and drug-naïve (FEDN) schizophrenia.

## Materials and Methods

### Participants

We recruited a total of 90 patients (48 men and 42 women) from Beijing Huilongguan Hospital, one of the largest public psychiatric hospitals in China. We diagnosed patients with schizophrenia up admission using the Diagnostic and Statistical Manual of Mental Disorders, Fourth Edition (DSM-IV), and then we confirmed the diagnosis 12 weeks late (35). We defined patients as FEDN based on a previous study by Lieberman et al. (36). Inclusion criteria included: (1) age between 16 and 55 years; (2) acute episodes of DSM-IV schizophrenia at admission; (3) duration of symptoms ≤ 60 months; (4) never received any antipsychotic, antidepressant, or any other psychoactive treatment; (5) score ≥ 4 as assessed by the Clinical Global Impression-Severity scale (CGI-S); and (6) able to provide written informed consent. Upon admission, all patients were physically examined and provided a complete medical history. Among them, those meeting the following criteria were excluded: (1) current major physical illness; (2) personal history of neurological disorders; (3) history of alcohol or drug dependence in addition to smoking; and (4) inability to provide signed consent.

While the patients were being recruited, 60 healthy controls (31 men and 29 women) were recruited in the local community. Unstructured interviews and a personal and family psychiatric history were used to determine their current mental status. Individuals with a history of mental illness were barred from participating.

The research protocol was approved by the Institute Review Board, Beijing Huilongguan hospital. To be a part of the study, each participant had to complete an informed consent form.

### Clinical Measures

The PANSS and the 17-item Hamilton Depression Scale (HAMD-17) were applied to measure psychopathology and depressive symptoms, respectively. Additionally, the clinical severity was assessed using the Clinical Global Impression Inventory (CGI). The PANSS, HAMD-17, and CGI evaluations were administered by four psychiatrists who had been trained in their usage prior to the commencement of the research to assure the reliability and consistency of the scales. After training, the correlation coefficient between investigators while repeatedly scoring the PANSS, HAMD-17, and CGI was more than 0.80.

The 17-item Hamilton Rating Scale for Depression (HAMD) was applied to assess depressive symptoms, with nine items scored from 0 (absent) to 2 (symptom-specific severity descriptors), and eight items scored from 0 (absent) to 4 (severe). Items 8, 9, and 11 were based on observation of the patient; the remaining items are assessed based on the patient’s own verbal narrative; item 1 required a combination of both. There were two other points to note: item 7 required information from the patient’s family, and item 16 could be assessed based on the patient’s complaint and information from the patient’s family. According to a previous study, we defined schizophrenia patients with depressive symptoms as having a total HAMD-17 score ≥ 8, while those without depressive symptoms had a total score ≤ 7 (37).

### Blood Sampling and Serum Brain-Derived Neurotrophic Factor Measurements

Serum samples were collected from participants between 7:00 and 9:00 am after an overnight fast. In order to conduct the tests, samples were obtained on the same day as the clinical evaluation and separated, aliquoted, and stored at −70°C. A commercially available kit (R&D Systems, Beijing, China) was used to measure serum BDNF levels using a sandwich enzyme-linked immunosorbent assay. These assays are detailed in full in our prior reports (38, 39). The same technician, who had no knowledge of the clinical circumstances, measured all serum samples. Meanwhile, the principal investigator applied codes to identify all participants until all biochemical analyses were completed. The inter- and intra-assay coefficients of variation were 7 and 5%, respectively.

### Statistical Analysis

First, the Kolmogorov-Smirnov one-sample test and the Q-Q plot were used to verify normality. For normally distributed numerical variables, mean ± standard deviation were used, whereas numbers and percentages were used for categorical variables. Demographic factors were compared using analysis of variance (ANOVA) for continuous variables and chi-square analysis for categorical variables. Demographic factors were compared using analysis of variance (ANOVA) for continuous variables and chi-square analysis for categorical variables. Furthermore, demographic parameters were taken into account as covariates in the comparison of serum BDNF levels between patients and healthy controls. The relationship between the variables was assessed with the Pearson’s product moment correlation method. In addition, further multiple linear regression analysis was used to explore correlations between the variables. All dependent variables were normally distributed and there was no evidence of multicollinearity.

We used SPSS (IBM SPSS 24.0, SPSS Inc.) and GraphPad Prism 8 (GraphPad Software Inc.) for all our analyses, with the significance level α set at *p* < 0.05 (two-tailed test).

## Results

### Comparison of Patients and Healthy Controls

As shown in Table 1, each participant’s demographic, and clinical data were summarized. There was no significant sex difference between the patients with FEDN schizophrenia and healthy controls. However, the patients were younger than the healthy controls (*p* < 0.05).

**TABLE 1:** Socio-demographic and clinical characteristics of the participants.

Variable	Schizophrenia (*n* = 90)	Healthy controls (*n* = 60)	*F* or *X*^2^	*P*-value
Sex, M/F	48/42	31/29	0.040	0.841
Smokers, *N* (%)	27 (30.0%)	22 (36.7%)	1.002	0.317
Heredity				
With, *N* (%)	27 (30.0%)			
Without, *N* (%)	63 (70.0%)			
Age, years	29.1 ± 9.6	33.0 ± 9.8	5.77	0.017*
BDNF, ng/ml	9.1 ± 4.6	12.1 ± 1.7	21.076*^a^*	0.000***

*BDNF, brain-derived neurotrophic factor.*

*^a^Analysis of covariance (ANCOVA).*

*p < 0.05; ***p < 0.001.

In the analysis of covariance, we included demographic factors such as age, sex, and smoking status as covariates. Serum BDNF levels in patients with FEDN schizophrenia were significantly lower than those in healthy controls (*p* < 0.001).

### Clinical and Demographic Data and Serum Brain-Derived Neurotrophic Factor of the Patients With and Without Depressive Symptoms

The Socio-demographic and clinical characteristics of patients with and without depressive symptoms are shown in Table 2. An analysis of variance (ANOVA) was used to compare the demographic features of these two subgroups. There was no significant difference in sex, age, years of education, smoking status, or heredity between patients with and without depressive symptoms (all *p* > 0.05).

**TABLE 2:** Socio-demographic and clinical characteristics of the patients.

Variable	Schizophrenia		*F* or *X*^2^	*P*-value
	Non-depression (*n* = 30)	Depression (*n* = 60)		
Sex, M/F	16/14	32/28	0.000	1.000
Smokers, *N* (%)	5 (18.5%)	22 (81.5%)	3.810	0.051
Heredity			0.238	0.626
With, *N* (%)	10 (37.0%)	17 (63.0%)		
Without, *N* (%)	20 (31.7%)	43 (68.3%)		
Age, years	27.6 ± 8.9	29.9 ± 9.9	1.170	0.282
Age of onset, years	25.6 ± 9.1	27.8 ± 10.2	1.047	0.309
Education, years	12.1 ± 4.3	12.0 ± 3.2	0.043	0.836
BDNF, ng/ml	7.5 ± 2.7	9.9 ± 5.3	4.307^a^	0.042*
PANSS score, mean ± SD				
Positive symptoms	23.7 ± 5.2	26.6 ± 6.5	3.302^a^	0.073
Negative symptoms	18.0 ± 5.5	18.6 ± 7.9	0.003^a^	0.955
General symptoms	33.0 ± 5.5	43.1 ± 12.2	15.671^a^	0.000***
Total score	74.8 ± 10.0	88.3 ± 20.7	8.731*^a^*	0.004***

*BDNF, brain-derived neurotrophic factor; PANSS, Positive and Negative Syndrome Scale.*

*p < 0.05; ***p < 0.001.

*^a^Analysis of covariance (ANCOVA).*

Moreover, no significant relationship was observed between serum BDNF levels and age, sex, education, smoking status, heredity, or age of onset in patients with and without depressive symptoms (all *p* > 0.05; Table 3).

**TABLE 3:** Correlations between BDNF and socio-demographic characteristics and PANSS scores in schizophrenia patients^a^.

	Schizophrenia (*n* = 90)
Sex	0.041 (0.744)
Age	−0.099 (0.424)
Age of onset	−0.133 (0.283)
Education	−0.31 (0.802)
Smoking status	0.026 (0.837)
Heredity	0.207 (0.093)
PANSS score	
Negative symptoms	0.178 (0.150)
General symptoms	0.238 (0.052)

*^a^Values are shown as r (p value).*

In addition, analysis of covariance (ANCOVA) was conducted, considering smoking status as a covariate. Serum BDNF levels were shown to be higher in individuals with depressive symptoms compared to those without such symptoms (9.9 ± 5.3 vs. 7.5 ± 2.7 ng/mL; *p* < 0.05).

### Serum Brain-Derived Neurotrophic Factor Levels and Psychopathological Symptoms of Patients

As shown in Table 2, patients without depression showed the following mean scores on PANSS: positive subscore, 23.7 ± 5.2; negative subscore, 18.0 ± 5.5; general psychopathology score, 33.0 ± 5.5, and PANSS total score, 74.8 ± 10.0. For patients with depression: positive subscore, 26.6 ± 6.5; negative subscore, 18.6 ± 7.9; general psychopathology score, 43.1 ± 12.2, and PANSS total score, 88.3 ± 20.7.

Smoking status was taken into account as a covariate in the analysis of covariance (ANCOVA). There were significant differences in PANSS general psychopathology (*p* < 0.001) and PANSS total scores (*p* = 0.001) between patients with and without depressive symptoms.

**FIGURE 1:**
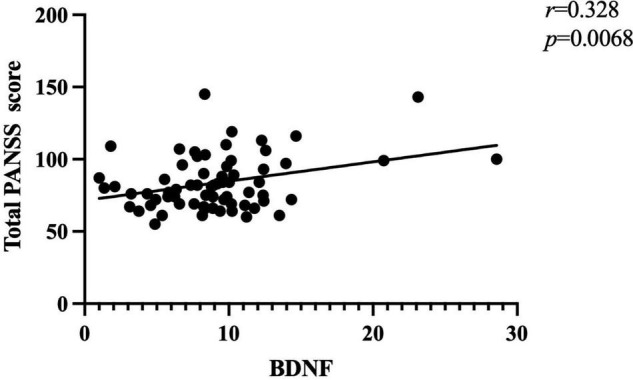
There was a positive association between BDNF levels and total PANSS score (*r* = 0.328, *p* < 0.01) in patients with FEDN schizophrenia.

**FIGURE 2:**
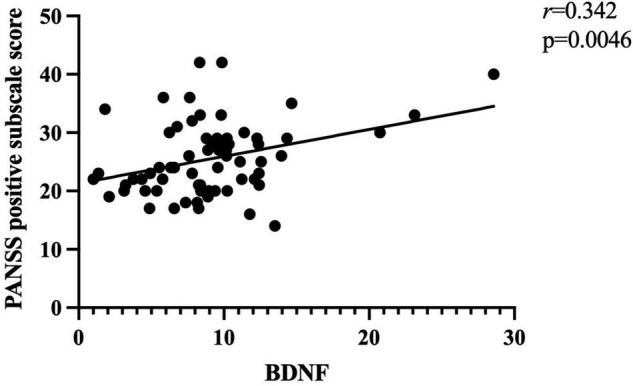
There was a positive association between BDNF levels and PANSS positive subscore (*r* = 0.342, *p* < 0.01) in patients with FEDN schizophrenia.

Further correlation analysis showed a significant association between BDNF levels and PANSS total scores (*r* = 0.328, *p* < 0.01; Figure 1). There was also a significant association between BDNF levels and positive symptom score (*r* = 0.342, *p* < 0.01; Figure 2) and further multiple linear regression affirmed this result (β = 0.342, 95% CI: 0.018–0.425, *p* = 0.005). However, no significant association was found between BDNF and other subscales (all *p* > 0.05; Table 3).

### Correlation Between Brain-Derived Neurotrophic Factor and Depressive Symptoms in Patients With Schizophrenia

Correlation analyses did not show a significant association between BDNF levels and HAMD scores in all patients with schizophrenia (*p* > 0.05; Table 3).

## Discussion

In our current study, there were four main findings. (1) Serum BDNF levels were significantly lower in patients with FEDN schizophrenia than in healthy controls. (2) Serum BDNF levels were higher in FEDN schizophrenia patients with depressive symptoms than in patients without depressive symptoms. (3) There was a significant association between serum BDNF levels and positive symptoms (*p* < 0.01). (4) There was no significant association between serum BDNF levels and HAMD scores.

### Serum Brain-Derived Neurotrophic Factor Levels in Patients With First-Episode and Drug-Naïve Schizophrenia

In this study, serum BDNF levels were lower in patients with FEDN schizophrenia than in healthy controls, confirming our prior studies (12, 15) and consistent with other studies in patients with first-episode schizophrenia (14, 40), and chronic schizophrenia (41–43), albeit not all (44). Levels of BDNF in the hippocampus and prefrontal cortex have been demonstrated to be lower in patients with schizophrenia (45). Moreover, several studies have shown a substantial positive correlation between peripheral BDNF levels and central nervous system BDNF levels (46, 47). According to previous studies, peripheral BDNF from platelets, immune cells, and vascular endothelial cells may cross the blood-brain barrier (48). Thus, decreased serum BDNF levels may indicate neuropathological alterations in the brain, confirming the neurodevelopment hypothesis, which claims that schizophrenia is the outcome of pathological processes that begin during prenatal and postnatal central nervous system development (49).

For the time being, there are still no conclusive results regarding the BDNF levels in the serum of schizophrenia patients with depressive symptoms. Our study showed that schizophrenia patients with depressive symptoms had higher serum BDNF levels than those without depressive symptoms, which is consistent with previous studies (34), but contradicts the findings of some investigations (50, 51). Many factors may play a role in these differences, such as duration of untreated psychosis, age of onset, duration of illness, test material (plasma versus serum), and different ethnicity of the subjects (52, 53). Furthermore, numerous studies on inflammatory and oxidative stress markers in schizophrenia have demonstrated an association between unmediated first episode psychosis and systemic inflammation and oxidative stress, as indicated by elevated levels of cytokines and oxidative stress markers in peripheral blood (54–57). In addition, BDNF has been shown to have a compensatory function by reducing oxidative damage to lipids and proteins via modulating antioxidant defenses when rats were exposed to chronic unpredictable moderate stress (58). Therefore, higher levels of BDNF in FEDN schizophrenia patients with depressive symptoms may be a compensatory response to reduced antioxidant status and pro-inflammatory imbalance (59) in patients with first-episode schizophrenia (54, 60).

### Association Between Brain-Derived Neurotrophic Factor and Clinical Symptoms

We found that patients without depressive symptoms performed lower on the PANSS total score and general subscore than patients with depressive symptoms. Additionally, we observed no difference in the positive subscore between the groups of patients, which is consistent with Calderon-Mediavilla’s previous study (61). There was also no difference in the negative subscore. Given that negative symptoms are less frequent in the initial year of schizophrenia, and peak between 2 and 5 years following the first admission, it is understandable that no difference was detected (62).

Another finding of this study was the significant correlation between serum BDNF levels and positive symptoms. In several previous studies, BDNF levels have been associated with both positive symptoms (63, 64) and negative symptoms (65). According to previous findings, BDNF and the dopaminergic system are intimately linked. There have been reports that BDNF and its receptor TrkB were expressed in the midbrain dopamine (DA) circuit (66). There is also further evidence to suggest that BDNF may enhance the release of DA (67). Meanwhile, several theories have argued that uncoordinated DA signaling is to blame for positive symptoms such as delusions and hallucinations (68, 69). Therefore, we propose that the positive correlation between BDNF and positive symptom score observed in the current research was due to an aberrant interaction between the BDNF and DA systems, which warrants further investigation.

In the current study, no significant association was noted between serum BDNF levels and HAMD scores, which is consistent with some previous studies (70–72), but is not definitive (73). Nevertheless, to our knowledge, the current study is the first to include patients with first-episode schizophrenia as a study population. The discrepancy may be due to the sampling of patients at different stages of disease progression. Also, the relationship between serum BDNF levels and the severity of depression is complicated by many factors that cannot be ignored, such as seasonality, physical activity, and different methods of BDNF measurement (74–76). Accordingly, to determine how serum BDNF levels influence depressive symptoms in patients with FEDN schizophrenia, more research needs to be done.

Our study had several limitations. First, case-control studies cannot be used to draw conclusions regarding the relationship between serum BDNF levels and depressive symptoms in patients with FEDN schizophrenia. Second, patients in the current study were inpatients recruited at a psychiatric hospital in Beijing, with a relatively limited sample size, constraining the application of our results to outpatients and other settings before conclusive conclusions can be reached. Third, additional factors not investigated in this research, such as exercise, a history of suicide attempts, drug use other than smoking, and seasonality, may impact the prevalence or severity of depressive symptoms in patients with FEDN schizophrenia. Fourth, the HAMD-17 was not originally intended to evaluate depressive symptoms in patients with schizophrenia. The Calgary Schizophrenia Depression Inventory (77), which better distinguishes between depressive and negative symptoms of schizophrenia, could have been employed instead. Fifth, we needed to obtain blood samples from subjects in this study, but many healthy controls were reluctant to provide blood samples. Furthermore, there were design flaws in this study. Therefore, these factors resulted in unbalanced sample sizes between the schizophrenia and healthy control groups. We will conduct a follow-up study with a larger sample size and to ensure equal numbers in both groups. Therefore, to corroborate our present results, further longitudinal studies with larger study populations that control for confounding variables are required.

In summary, we conducted a clinical study to clarify the relationship between serum BDNF levels and depressive symptoms in patients with FEDN schizophrenia. Patients with depressive symptoms in the early stages of schizophrenia were shown to have higher levels of BDNF in their serum. In addition, low serum BDNF levels were associated with positive symptoms of schizophrenia but not with depressive symptoms. The study’s relatively small sample size limited the extension of the findings. In follow-up studies, we will include more samples to confirm the findings in this study. Further longitudinal and prospective investigations may offer new venues for exploring the relationship between serum BDNF levels and depressive symptoms in patients with FEDN schizophrenia.

## Data Availability Statement

The raw data supporting the conclusions of this article will be made available by the authors, without undue reservation.

## Ethics Statement

The studies involving human participants were reviewed and approved by the Institute Review Board of Beijing Huilongguan Hospital. The patients/participants provided their written informed consent to participate in this study.

## Author Contributions

YW was responsible for study design, statistical analysis, and wrote the original draft. RY, YY, RP, SW, HW, YZ, and XF were involved in data curation, visualization, and investigation. NY, RL, JZ, SZ, XLZ, XL, ZL, and XBZ were responsible for the evolution of ideas, supervision, and review and editing of the manuscript. XD and XYZ participated in funding acquisition, conceptualization, resources, proposal writing, and editing the manuscript. All authors contributed to and have approved the final manuscript.

## Conflict of Interest

The authors declare that the research was conducted in the absence of any commercial or financial relationships that could be construed as a potential conflict of interest.

## Publisher’s Note

All claims expressed in this article are solely those of the authors and do not necessarily represent those of their affiliated organizations, or those of the publisher, the editors and the reviewers. Any product that may be evaluated in this article, or claim that may be made by its manufacturer, is not guaranteed or endorsed by the publisher.
